# Long-Term Administration of Dehydroepiandrosterone Accelerates Glucose Catabolism *via* Activation of PI3K/Akt-PFK-2 Signaling Pathway in Rats Fed a High-Fat Diet

**DOI:** 10.1371/journal.pone.0159077

**Published:** 2016-07-13

**Authors:** Jian Kang, Chongyang Ge, Lei Yu, Longlong Li, Haitian Ma

**Affiliations:** Key Laboratory of Animal Physiology and Biochemistry, College of Veterinary Medicine, Nanjing Agricultural University, Nanjing, China; East Tennessee State University, UNITED STATES

## Abstract

Dehydroepiandrosterone (DHEA) has a fat-reducing effect, while little information is available on whether DHEA regulates glucose metabolism, which would in turn affect fat deposition. To investigate the effects of DHEA on glucose metabolism, rats were administered a high-fat diet containing either 0 (HCG), 25 (HLG), 50 (HMG), or 100 (HHG) mg·kg^-1^ DHEA per day *via* gavage for 8 weeks. Results showed that long-term administration of DHEA inhibited body weight gain in rats on a high-fat diet. No statistical differences in serum glucose levels were observed, whereas hepatic glycogen content in HMG and HHG groups and muscle glycogen content in HLG and HMG groups were higher than those in HCG group. Glucokinase, malate dehydrogenase and phosphofructokinase-2 activities in HMG and HHG groups, pyruvate kinase and succinate dehydrogenase activities in HMG group, and pyruvate dehydrogenase activity in all DHEA treatment groups were increased compared with those in HCG group. Phosphoenolpyruvate carboxykinase and glycogen phosphorylase mRNA levels were decreased in HMG and HHG groups, whereas glycogen synthase-2 mRNA level was increased in HMG group compared with those in HCG. The abundance of *Glut2* mRNA in HMG and HHG groups and *Glut4* mRNA in HMG group was higher than that in HCG group. DHEA treatment increased serum leptin content in HMG and HHG groups compared with that in HCG group. Serum insulin content and insulin receptor mRNA level in HMG group and insulin receptor substrate-2 mRNA level in HMG and HHG group were increased compared with those in HCG group. Furthermore, *Pi3k* mRNA level in HMG and *Akt* mRNA level in HMG and HHG groups were significantly increased than those in HCG group. These data showed that DHEA treatment could enhance glycogen storage and accelerate glucose catabolism in rats fed a high-fat diet, and this effect may be associated with the activation of PI3K/Akt-PFK-2 signaling pathway.

## Introduction

Obesity poses a very serious threat to human health [1, 2], and it is associated with a number of metabolic diseases, such as chronic diabetic hyperglycemia, diabetes mellitus, hypertension, and fatty liver disease [3–6]. These diseases affect millions of individuals who must carefully control their blood glucose levels to prevent diabetes-related complications [6]. Increased intake of calorie-rich foods and a sedentary lifestyle are the main causes of obesity in humans worldwide [7]. In addition to exercise, healthy foods and food ingredients may be a practical way to control body weight and fat accumulation [8]. Although numerous studies have focused on various approaches to reduce body weight and control fat accumulation *via* administration of bioactive compounds [9–12], recently, there has been increasing concern on obesity associated with glycometabolism [4, 5, 13, 14].

Dehydroepiandrosterone (DHEA), a naturally occurring steroid, is mainly secreted by the adrenal cortex [15]. One characteristic of DHEA production is its age-associated production [16]; its aged-related decline has attracted attention with regard to physical health [17, 18]. Currently, DHEA is commercially available as a non-prescription nutritional supplement [19]. The administration of DHEA reduces body weight gain and visceral fat accumulation [11, 20]. Our laboratory previously showed that DHEA accelerated lipid catabolism by the activating of the cAMP-PKA signaling system [21], which will induced the expression of relevant genes expression [22]. It is well known that there is a close relationship between glucose metabolism and fat metabolism in the body. Administration of DHEA, a potential therapy for weight loss and fat accumulation reduction, may be a practical way to reduce body weight and excessive fat in humans or animals. However, little information is available to assess whether DHEA regulates glucose metabolism, which would in turn affect body weight and fat deposition.

Therefore, the present study was conducted to investigate the effects of the long-term administration of DHEA on glucose metabolism and its consequence in rats fed a high-fat diet. This information will deepen our understanding of the mechanisms driving DHEA and verify it as a nutritional supplement to control body weight and to curb obesity-related diseases.

## Materials and Methods

### Animals and dietary treatment

**Fig 1:**
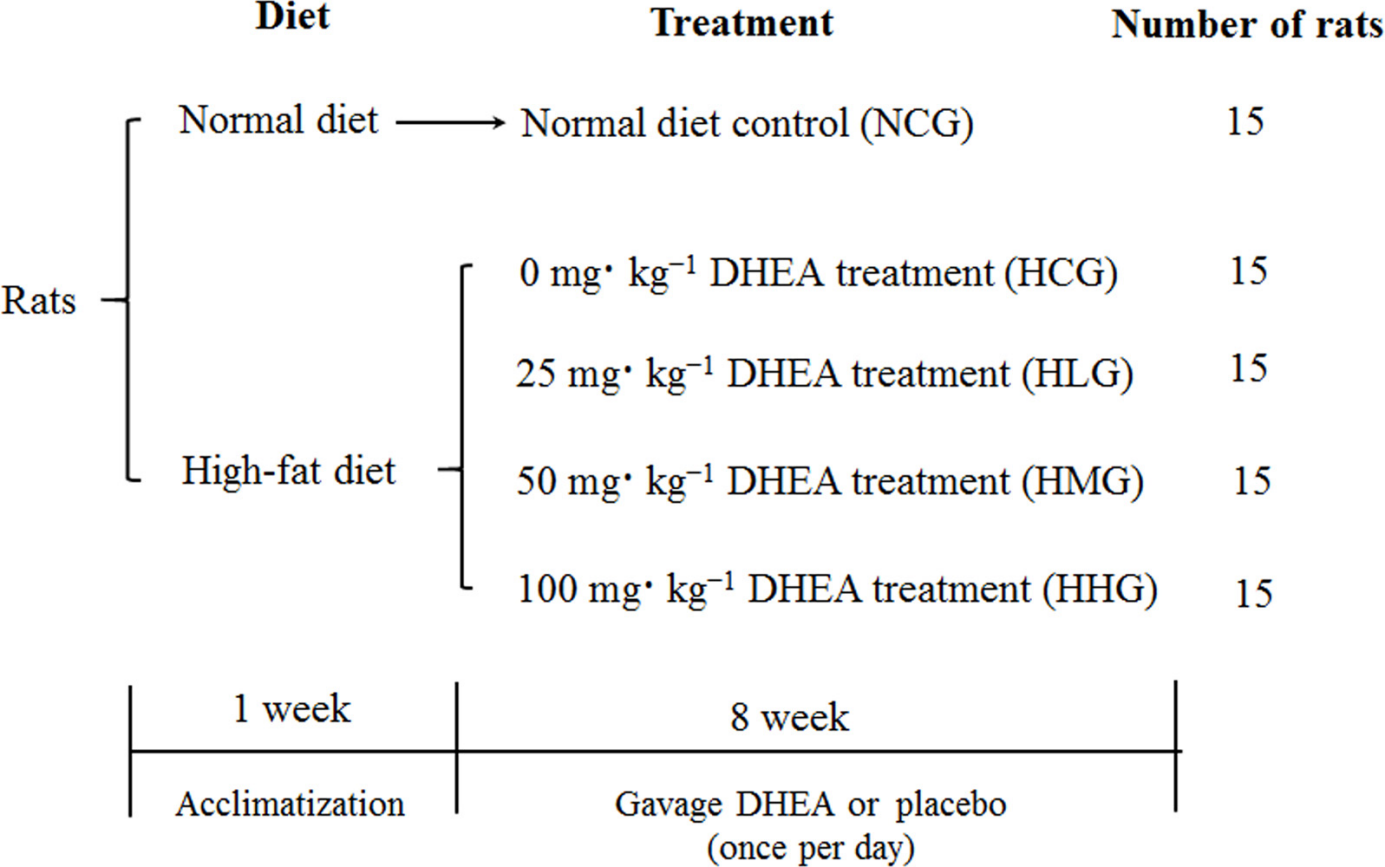
Experimental design.

Two-month-old Sprague Dawley rats weighing 200 ± 20 g were purchased from the Experimental Animal Center of the Jiangsu University (China). All animal handling procedures were performed in strict accordance with the Care and Use of Laboratory Animals central of the Nanjing Agricultural University guidelines. The protocol was approved by the Institutional Animal Care and Use Committee of the Nanjing Agricultural University. Rats were housed individually under a constant temperature of 25°C and humidity ranged between 50–60%, with a 12 h light: 12 h dark cycle. Animals were treated as indicated in Fig 1. Briefly, after one week of acclimatization, 75 rats were randomized into five groups: normal diet control group (NCG), high-fat diet control group (HCG), high-fat diet with low dose DHEA group (HLG), high-fat diet with medium dose DHEA group (HMG), and high-fat diet with high dose DHEA group (HHG). Rats in the NCG group were fed a normal diet, whereas those in the other groups were fed a high-fat diet with the corresponding DHEA treatment (purchased from Changzhou Jiaerke Pharmaceuticals Group Corp., and dissolved in 1% DMSO) *via* gavage at 0, 25, 50, and 100 mg·kg^−1^ body weight (for HCG, HLG, HMG, and HHG, respectively) once per day for 8 weeks. The NCG group rats received an equal volume of placebo each day for eight weeks. The normal diet (3.57 kcal·g^-1^ formula comprising, 24.6% protein, 16.4% fat, and 59% carbohydrates; GB14924, 3–2010, China) and high-fat diet (4.45 kcal·g^-1^ formula comprising, 18% protein, 44% fat, and 38% carbohydrates) were provided by the Jiangsu Xietong Biotechnology Institution (China). At the end of the experiment, food was removed for the final 12 hours, and then the rats were anesthetized with ether and sacrificed by decapitation. Blood samples were allowed to clot at 4°C and were then centrifuged at 1520 × g for 20 min before harvesting the serum. The serum, liver and muscle samples were collected and kept at -70°C until further analysis.

### Measurement of serum glucose and glycogen content

Serum glucose, hepatic glycogen, and muscle glycogen contents were measured using commercial kits according to the manufacturers’ protocols (Nanjing Jiancheng Biotechnology Institution, China).

### Measurement of key enzyme activity during glycometabolism in the liver

The activities of glucokinase (GK), pyruvate kinase (PK), succinate dehydrogenase (SDH), and malate dehydrogenase (MDH) in the liver were measured using commercial kits according to the manufacturers’ protocols (Nanjing Jiancheng Biotechnology Institution, China). The activities of pyruvate dehydrogenase (PDH, E1) and phosphofructokinase-2 (PFK-2) in the liver were measured using ELISA kits according to the manufacturers’ protocols (Shanghai Lengton Bioscience Co., China).

### Measurement of serum hormone content

Serum insulin and leptin contents were measured using radioimmunoassay kits according to the manufacturers’ protocols (Beijing North Institute of Biotechnology, China). The intra- coefficients of variation for all hormone detection kits were less than 10%, and inter- coefficients of variation were less than 15%.

### Determination of glucose metabolism-related factor gene mRNA levels by real-time PCR

Total RNA was extracted from liver and muscle samples using the TRIZOL reagent (Takara, Japan) according to the manufacturer’s protocol. Total RNA (1 μg) was reverse transcribed into cDNA using the Superscript II kit (Invitrogen, USA), according to the manufacturer’s recommendations. An aliquot of cDNA sample was mixed with 25 μL SYBR Green PCR Master Mix (TaKaRa, Japan) in the presence of 10 pmol each of forward and reverse primers for *β-actin* (used as an internal control), phosphoenolpyruvate carboxykinase (*Pepck*), glycogen phosphorylase (*Pygl*), glycogen synthase-2 (*Gys2*), insulin receptor (*Insr*), insulin receptor substrate-2 (*Irs2*), glucose transporter-2 (*Glut2*), phosphatidylinositol 3-kinase (*Pi3k*), and protein kinase B (*Akt*) in liver and glucose transporter-4 (*Glut4*) in muscle tissues. All samples were analyzed in duplicate in an ABI Prism 7300 Sequence Detection System (Applied Biosystems, Stockholm, Sweden) and programmed to conduct one cycle (at 95°C for 1 min) and 40 cycles (of 95°C for 30 s, 60°C for 30 s, and 72°C for 40 s). Fold change was calculated using the 2^-ΔΔCT^ method, and the relative amount of mRNA for each target gene was determined by calculating the ratio between each mRNA and the mRNA of β-actin [23, 24]. In our experimental system, DHEA treatment did not change the expression of β-actin. The primers used (Table 1) were designed using Primes Premier 5 and synthesized by Invitrogen Biological Company (China).

**Table 1:** Primer sequences for *β-actin* and other target genes.

Gene	GenBank accessionNumber	Primer sequences (5′-3′)	Orientation
*β-actin*	NM_031144	GATTACTGCCCTGGCTCCTA	Forward
		TCATCGTACTCCTGCTTGCT	Reverse
*Pi3k*	NM_053481	CGGCGTGACATGTAGGCTCTCA	Forward
		ACGGCCCGCACTGTAACCTAT	Reverse
*Akt*	NM_033230	TCAGTAGCATCCGGCAATTATC	Forward
		TGCTCAATCAAAGCCACAGTC	Reverse
*Pepck*	NM_198780	GCTGCCATGAGATCAGAGG	Forward
		AATCCGGGCCAGAGGAAC	Reverse
*Gys2*	NM_013089	TGGCCTCCAGCAAGTCAT	Forward
		TGGGATGTGGTTCAGGGA	Reverse
*Inrs*	NM_017071	GCAGAGACCCGTGTTGCGGT	Forward
		CCATCACTACCAGCATTGGCTGTCC	Reverse
*Glut2*	NM_012879	TGCTGGAAGAAGCGTATCAG	Forward
		GGCCAAGTAGGATGTGCCAG	Reverse
*Glut4*	NM_012751	TGTTGCGGATGCTATGGG	Forward
		CTGCGAGGAAAGGAGGGA	Reverse
*Irs2*	AF087674	CATCGTGAAGAAGGCATAGG	Forward
		GACCGGTGACGGCTGAACGG	Reverse

### Statistical analysis

All statistical analyses were performed with SPSS 17.0 for Windows (StatSoft, Inc., Tulsa, OK, USA), and results are expressed as means ± SE. A *t*-test was used to compare diet control groups, and a one-way analysis of variance (ANOVA) was used to compare the effects of DHEA concentrations within groups fed the same diet. Differences were considered significant at *P* < 0.05.

## Results

### Impact of DHEA on body weight, body mass index (BMI) and Lee’s index in rats fed a high-fat diet

**Fig 2:**
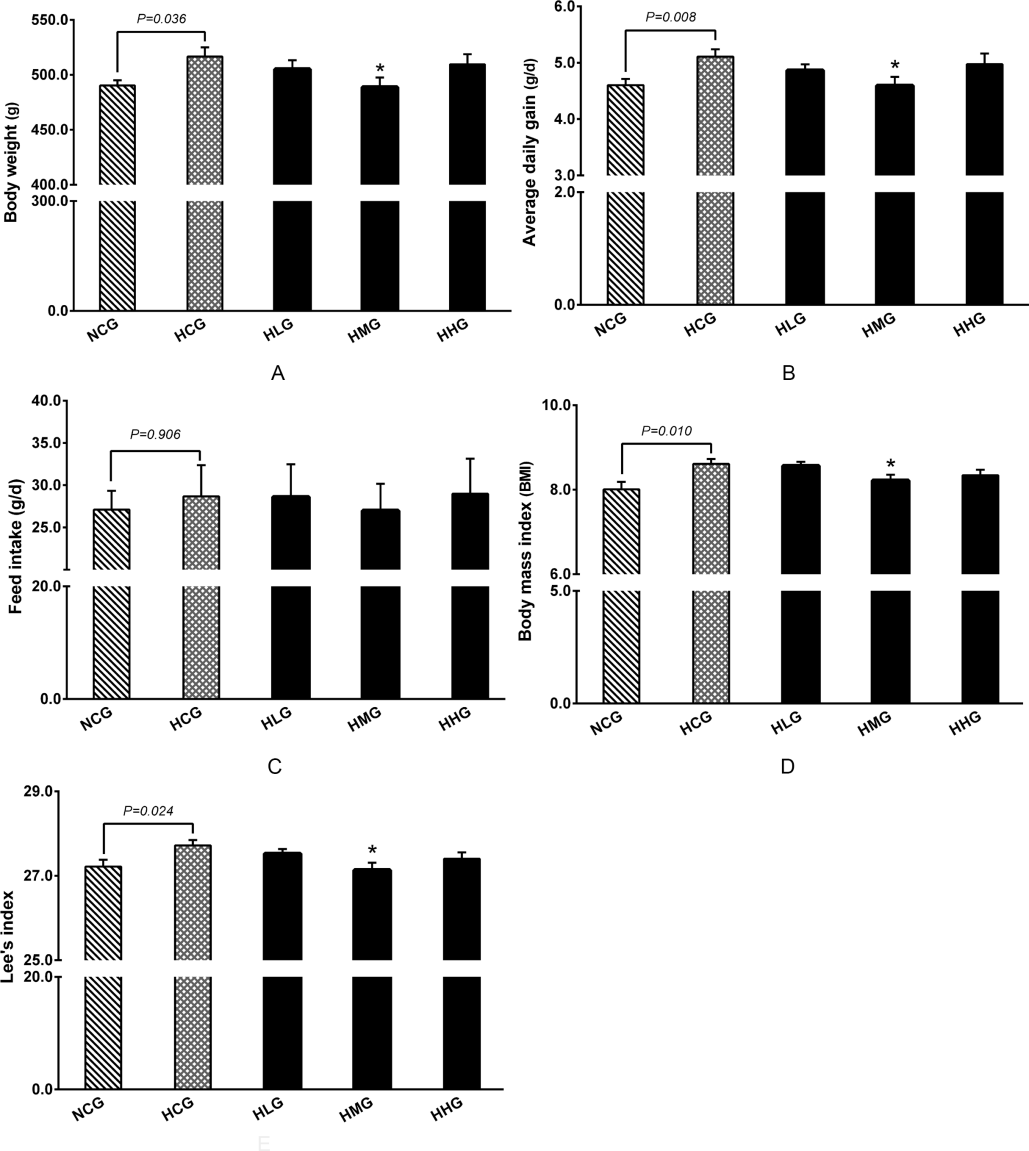
Effect of DHEA on body weight, Feed intake, BMI, and Lee’s index in rats fed a high-fat diet. A: Body weight; B: Average daily gain; C: Feed intake; D: Body mass index (BMI); E: Lee`s index. Data are expressed as means ± SE (n = 15). * *P* < 0.05, compared with the HCG group.

Body weight and average daily gain in the HCG group were significantly higher (*P* < 0.05) than in the NCG group (Fig 2). Consumption of the high-fat diet resulted in a greater degree of obesity among rats in the HCG group than in the NCG group (*P* < 0.05), as indicated by significantly higher BMI and Lee’s indices. No statistical differences were observed on feed intake in DHEA treatment groups compared with the HCG group (*P* > 0.05) (Fig 2C). Body weight and average daily gain were markedly reduced in the HMG group compared with the HCG group (*P* < 0.05) (Fig 2A and 2B). In addition, BMI and Lee’s indices were significantly lower in the HMG group than they were in the HCG group (*P* < 0.05) (Fig 2D and 2E).

### Effect of DHEA on serum glucose level and metabolism hormone concentration in rats fed a high-fat diet

**Fig 3:**
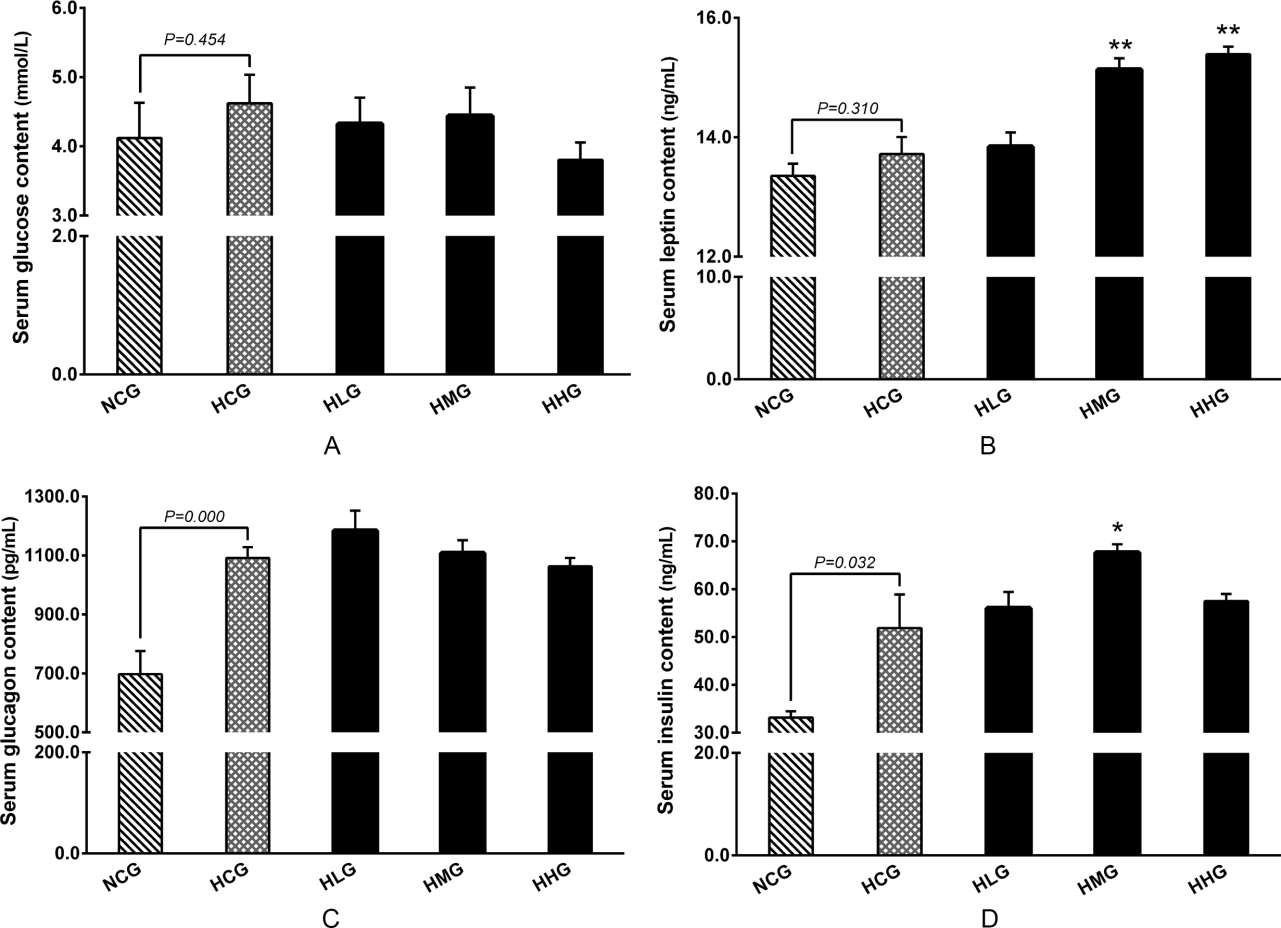
Effect of DHEA on glucose content and metabolic hormone in rats fed a high-fat diet. A: Glucose content; B: Leptin content; C: Glucagon content; D: Insulin content. Data are expressed as means ± SE (n = 15). ** *P* < 0.01 and * *P*< 0.05, compared with the HCG group.

No statistical differences were observed regarding the effect of DHEA on serum glucose and leptin contents between the HCG and NCG groups (*P* > 0.05), whereas serum glucagon and insulin contents were significantly higher in the HCG group than in the NCG group (*P* < 0.05) (Fig 3). Compared with the HCG group, DHEA treatment tended to decrease serum glucose level in rats fed a high-fat diet (*P* > 0.05) (Fig 3A). DHEA treatment significantly increased serum leptin content in the HMG and HHG groups compared with those in the HCG group (*P* < 0.01) (Fig 3B). No statistical differences were detected in glucagon content between DHEA treatment groups and HCG group (*P* > 0.05) (Fig 3C). Compared with the HCG group, serum insulin content in the HMG group was significantly higher (*P* < 0.05) (Fig 3D).

### Effect of DHEA on glycogen content and glycogen metabolism in rats fed a high-fat diet

**Fig 4:**
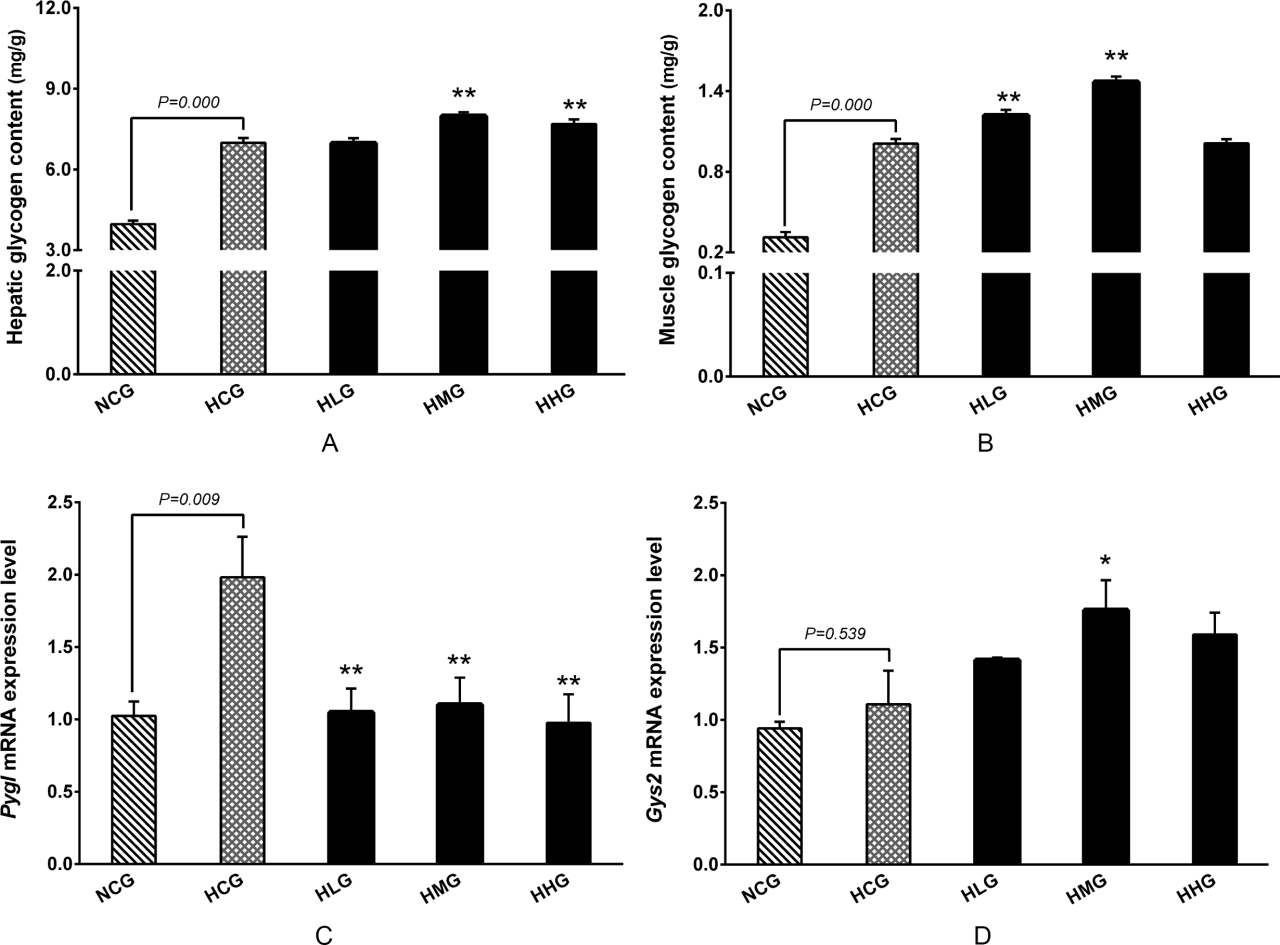
Effect of DHEA on glycogen contents and mRNA expression of key glycogen metabolism enzymes in rats fed a high-fat diet. A: Hepatic content; B: Muscle content; C: Glycogen phosphorylase (*Pygl*) mRNA level in liver; D: Glycogen synthase-2 (*Gys2*) mRNA level in liver. Data are expressed as means ± SE (n = 15). ** *P* < 0.01, compared with the HCG group.

Hepatic and muscle glycogen contents in the HCG group were significantly higher compared with the NCG group (*P* < 0.01) (Fig 4A and 4B). The hepatic glycogen contents were significantly higher in the HMG and HHG groups than that in the HCG group (*P* < 0.01) (Fig 4A). Compared with the HCG group, muscle glycogen contents in the HLG and HMG groups were significantly higher in the high-fat diet-fed rats (*P* < 0.01) (Fig 4B). In addition, *Pygl* mRNA levels were significantly higher in the HCG group than that in the NCG group (*P* < 0.01) (Fig 4C), whereas no significant difference was observed on *Gys2* mRNA level in the HCG group when compared to the NCG group (*P* > 0.05) (Fig 4D). Compared with the HCG group, administration of DHEA significantly decreased *Pygl* mRNA levels in rats fed a high-fat diet (*P* < 0.01) (Fig 4C). However, *Gys2* mRNA levels were significantly higher in the HMG group than that in the HCG group (*P* < 0.05) (Fig 4D).

### Effect of DHEA on glucose catabolism in rats fed a high-fat diet

**Fig 5:**
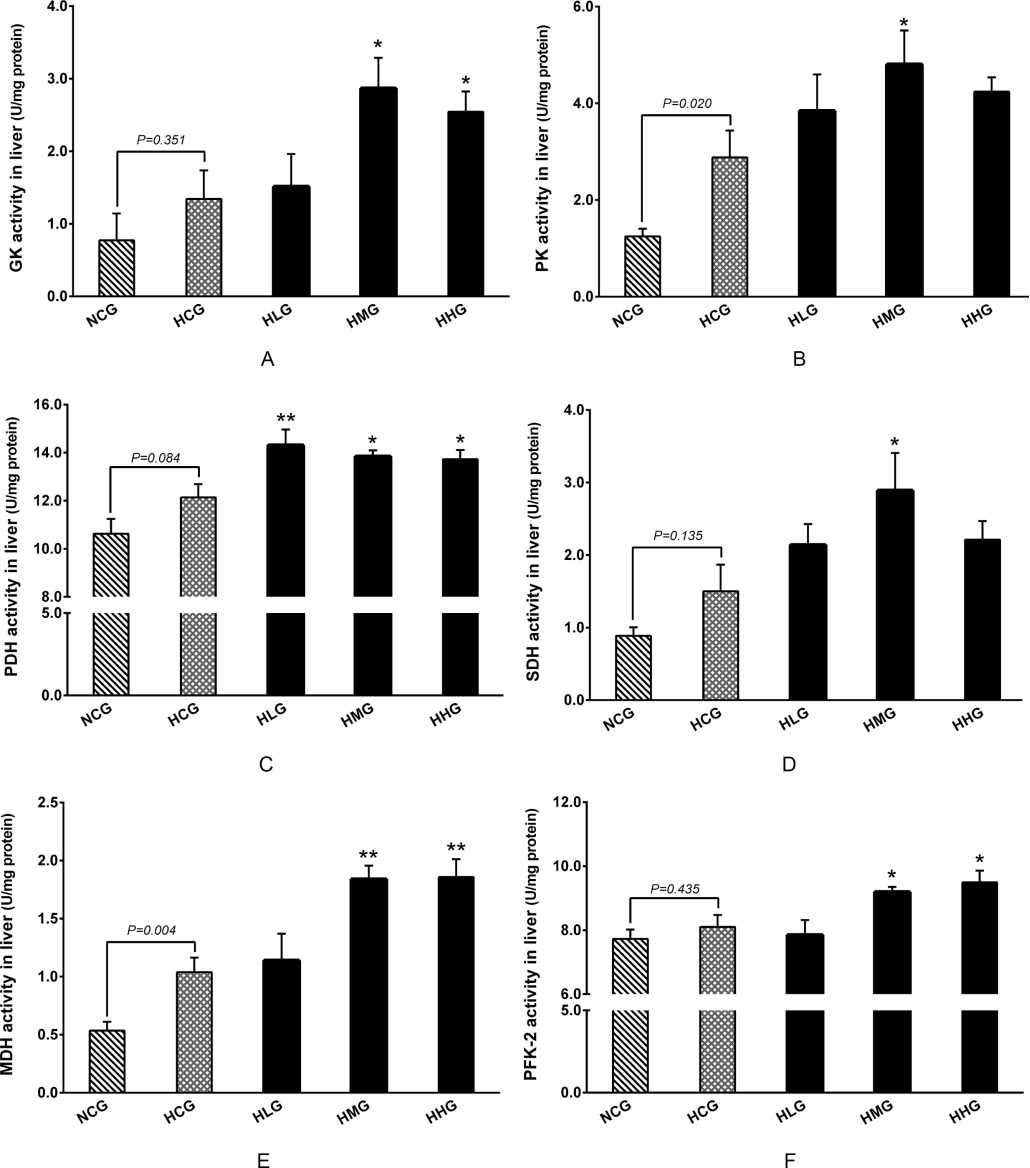
Effect of DHEA on key enzymes activities in the liver of rats fed a high-fat diet. A: Glucokinase (GK) activity; B: Pyruvate kinase (PK) activity; C: Pyruvate dehydrogenase (PDH) activity; D: Succinate dehydrogenase (SDH) activity; E: Malate dehydrogenase (MDH) activity; F: Phosphofructokinase-2 (PFK-2). Data are expressed as means ± SE (n = 15). ** *P* < 0.01 and * *P*< 0.05, compared with the HCG group.

No significant differences were observed in the GK, PDH (E1), SDH, and PFK-2 activities between the HCG and NCG groups (*P* > 0.05), while MDH and PK activities in the HCG group were significantly higher than those in the NCG group (*P* < 0.05) (Fig 5). The GK activity in the HMG and HHG groups (*P* < 0.05) (Fig 5A) and PK activity in the HMG group (*P* < 0.05) (Fig 5B) were significantly increased compared with those in the HCG group. Compared with the HCG group, administration of DHEA significantly increased PDH (E1) activity in rats fed high-fat diet (*P* < 0.05) (Fig 5C). Similarly, SDH activity in the HMG group (*P* < 0.05) (Fig 5D) and MDH activity in the HMG and HHG groups (*P* < 0.01) (Fig 5E) were significantly increased when compared with the HCG group. In addition, we found that PFK-2 activities were significantly increased in the HMG and HHG groups compared with that in the HCG group (*P* < 0.05) (Fig 5F).

### Effect of DHEA on gluconeogenesis in rats fed a high-fat diet

**Fig 6:**
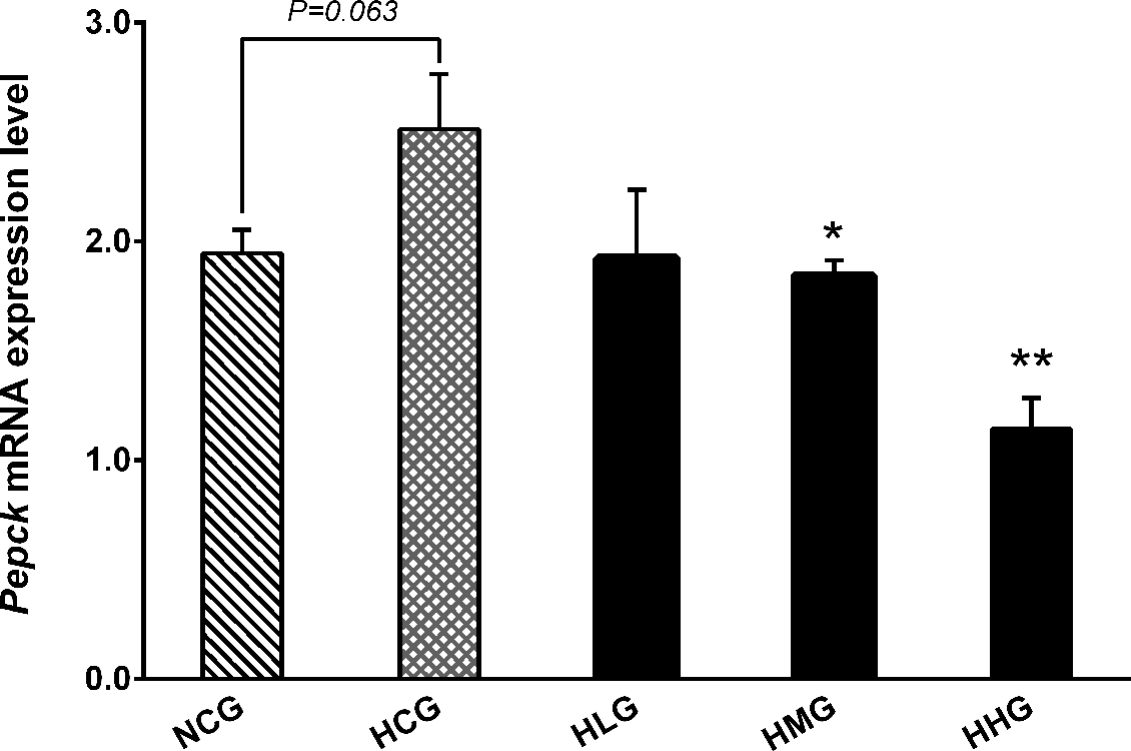
Effect of DHEA on phosphoenolpyruvate carboxykinase (*Pepck*) mRNA expression in the liver of rats fed a high-fat diet. Data are expressed as means ± SE (n = 15). ** *P* < 0.01 and * *P*< 0.05, compared with the HCG group.

No significant differences were observed in *Pepck* mRNA levels between the HCG and NCG groups (*P* > 0.05) (Fig 6). However, the *Pepck* mRNA levels were significantly decreased in the HMG (*P* < 0.05) and HHG (*P* < 0.01) groups compared with the HCG group (Fig 6).

### Effect of DHEA on glucose transporters in rats fed a high-fat diet

**Fig 7:**
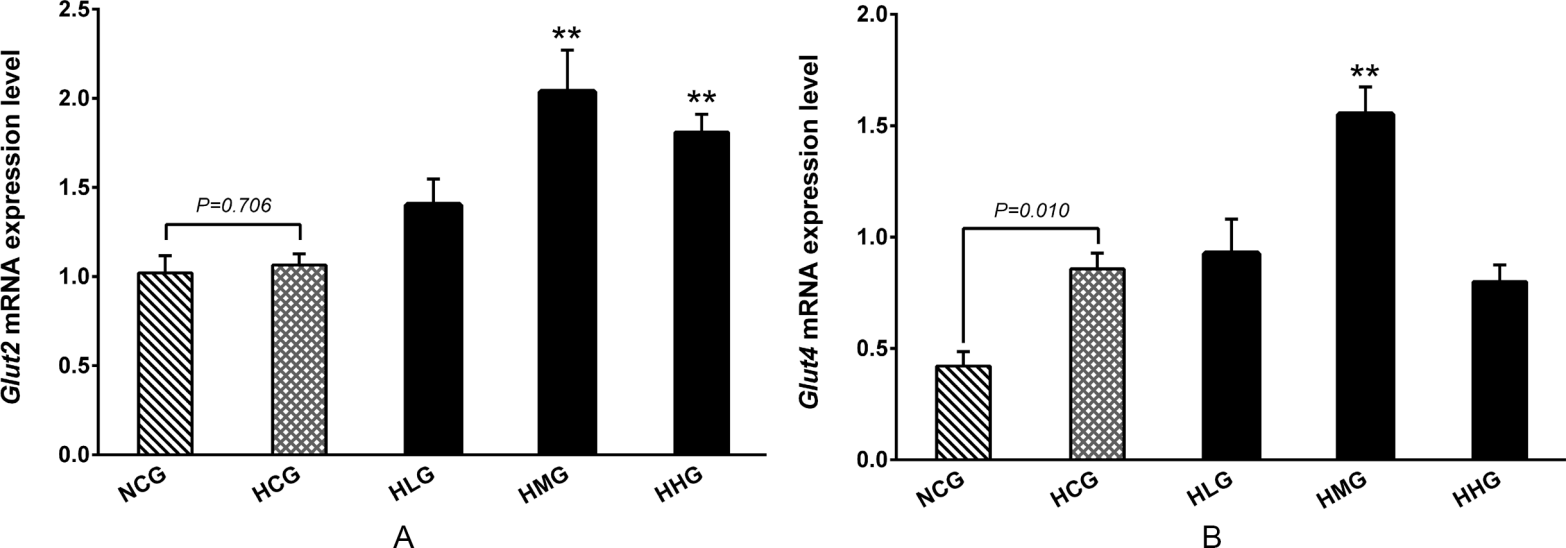
Effect of DHEA on glucose transporter mRNA expression in rats fed a high-fat diet. A: Glucose transporter-2 (*Glut2*) mRNA level in liver; B: Glucose transporter-4 (*Glut4*) mRNA level in muscle. Data are expressed as means ± SE (n = 15). ** *P* < 0.01, compared with the HCG group.

As shown in Fig 7, *Glut4* mRNA levels in the muscle were significantly higher in the HCG group than that in the NCG group (*P* < 0.01), whereas no differences were observed in *Glut2* mRNA level in the livers between the HCG and NCG groups (*P* > 0.05). DHEA treatment significantly increased *Glut2* mRNA level in the livers in the HMG and HHG groups compared with the HCG group (*P* < 0.01) (Fig 7A). In addition, the *Glut4* mRNA level in the HMG group was significantly higher in the muscle than that in the HCG group (*P* < 0.01) (Fig 7B).

### Effect of DHEA on insulin receptor and insulin receptor substrate mRNA levels in rats fed a high-fat diet

**Fig 8:**
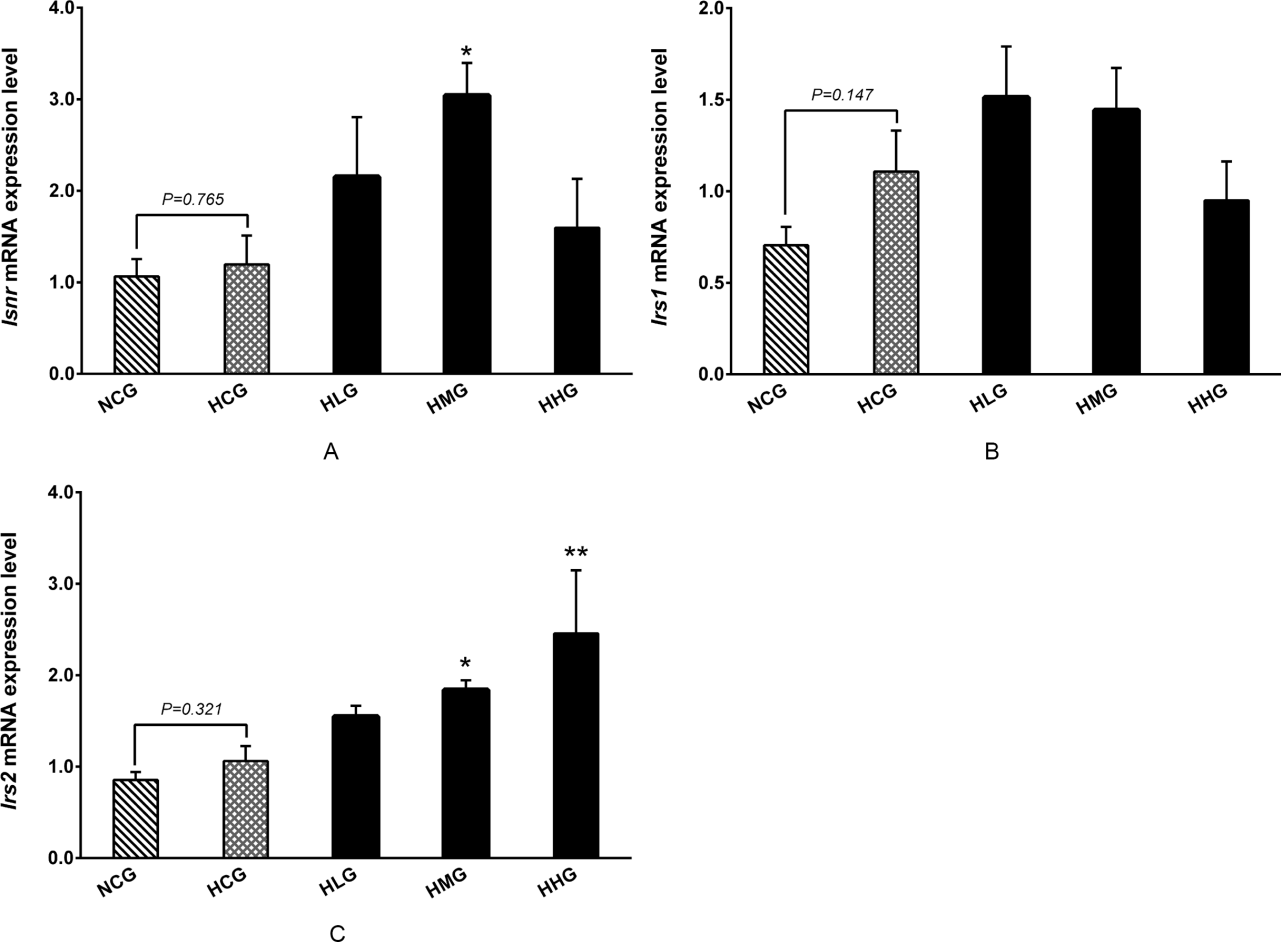
Effect of DHEA on insulin receptor and insulin receptor substrate mRNA expression in rats fed a high-fat diet. A: Insulin receptor (*Inrs*) mRNA level in liver; B: Insulin receptor substrate-1 (*Irs1*) mRNA level in muscle; C: Insulin receptor substrate-2 (*Irs2*) mRNA level in liver. Data are expressed as means ± SE (n = 15). ** *P* < 0.01 and * *P*< 0.05, compared with the HCG group.

No significant differences were observed on *Isnr* and *Irs2* mRNA levels in livers between the HCG and NCG groups (*P* > 0.05) (Fig 8). *Inrs* mRNA level was significantly higher in the HMG group than that in the HCG group (*P* < 0.05) (Fig 8A). In addition, no significant differences were observed in *Irs1* mRNA levels, whereas *Irs2* mRNA levels in the HMG and HHG groups were significantly higher than that in the HCG group (*P* < 0.05) (Fig 8B and 8C).

### Effect of DHEA on *Pi3k* and *Akt* mRNA levels in the liver of rats fed a high-fat diet

**Fig 9:**
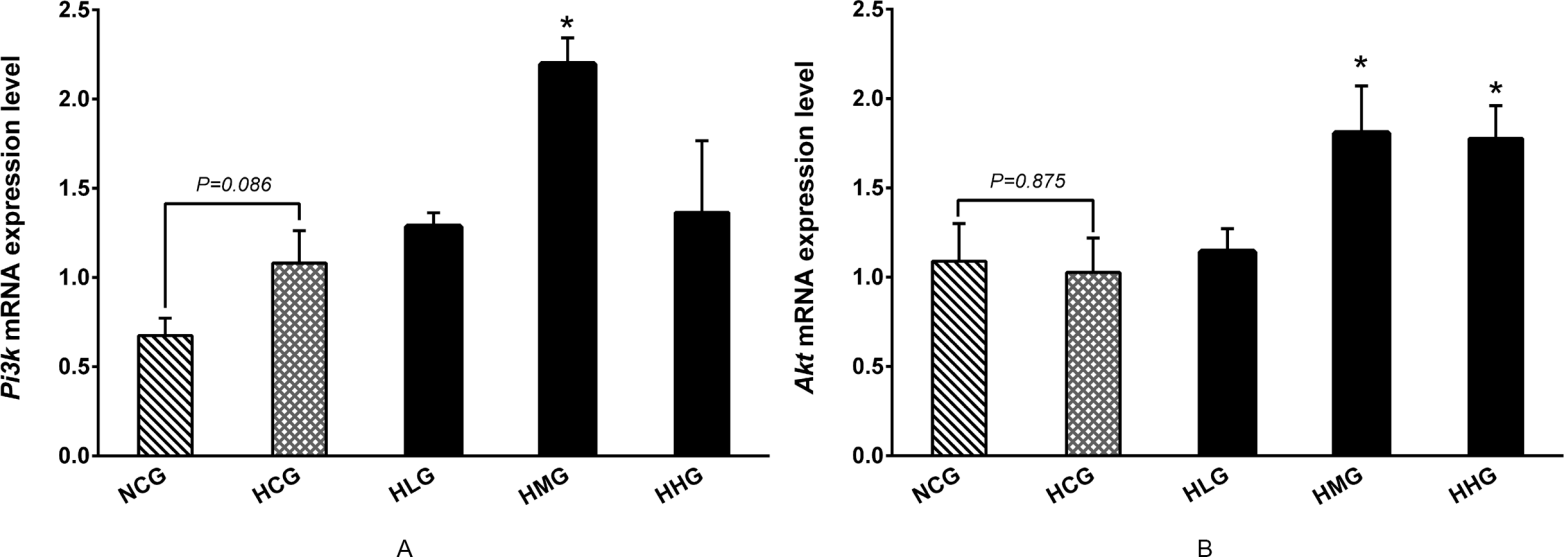
Effect of DHEA on phosphatidylinositol 3-kinase and protein kinase B mRNA expression in rats fed a high-fat diet. A: Phosphatidylinositol 3-kinaseand (*Pi3k*) mRNA level in liver; B: Protein kinase B (*Akt*) mRNA level in liver. Data are expressed as means ± SE (n = 15). ** *P* < 0.01 and * *P*< 0.05, compared with the HCG group.

As shown in Fig 9, no changes were observed in *Pi3k* and *Akt* mRNA levels between the HCG and NCG groups (*P* > 0.05). DHEA treatment significantly increased *Pi3k* mRNA levels in the HMG group compared with the HCG group (*P* < 0.05) (Fig 9A).The *Akt* mRNA levels in the HMG and HHG groups were significantly higher than that in the HCG group (*P* < 0.05) (Fig 9B).

## Discussion

Adiposity was determined by Lee’s index, which is defined as the cubic root of body weight in grams divided by the naso-anal length in millimeters multiplied by 10^4^ [10]. It has been shown that BMI and Lee’s index are highly correlated with obesity [10]. Our results showed that Lee’s index was significantly higher in rats under a high-fat diet than in rats on a normal diet. In addition, serum glucose content in rats on a high-fat diet increased by 12.15% relative to that in rats on a normal diet. Therefore, the high-fat diet in our experiment induced obesity in rats, which provides a model for further investigation of the preventative effect of DHEA on obesity in rats and its possible mechanisms.

Our results showed that long-term DHEA administration decreased body weight in rats on a high-fat diet. These results was consistent with a previous report showing long-term DHEA treatment results in the suppression of body weight gain in rodents [25], as well as with the results of Gansler *et al*. which demonstrated that long-term DHEA administration to lean or obese Zucker rats resulted in decreased body weight [26]. Many studies have found that DHEA administration reduces fat accumulation in chickens [22, 27, 28], rodents [29–32] and humans [18, 33]. DHEA has a fat-reducing effect, which may be accomplished through multiple mechanisms [34]. It is well known that fat accumulation relies on excess energy supply in the body. It was reported that DHEA treatment significantly promoted glucose conversion to glycogen, which could be one of the mechanisms controlling serum glucose levels [35]. Our results indicated that no differences were observed in glucose levels, whereas hepatic glycogen contents were markedly higher in the 50 and 100 mg·kg^−1^ DHEA treatment groups, and muscle glycogen contents were dramatically higher in the 25 and 50 mg·kg^−1^ DHEA treatment groups. Glycogen is synthesized in response to an increase in blood glucose concentration and processed into glucose to maintain blood glucose homeostasis. Glycogenesis and glycogenolysis during the diurnal cycle are mediated by glycogen synthase and glycogen phosphorylase (PYGL) [36]. Animal body usually express two isoforms of glycogen synthase, of which glycogen synthase-2 (GYS-2) appears to be the most important determinant of glycogen accumulation [37]. Administration of different doses of DHEA significantly decreased *Pygl* mRNA levels, and the 50 mg·kg^−1^ DHEA treatment significantly increased *Gys2* mRNA levels in rats on a high-fat diet. These results indicated that DHEA treatment increased glycogen content by enhancing the expression of glycogen synthase-2 and inhibiting the expression of glycogen phosphorylase.

Gluconeogenesis is a metabolic pathway that results in the generation of glucose from non-carbohydrate carbon substrates such as pyruvate, lactate, glycerol and glucogenic amino acids [38]. Phosphoenolpyruvate carboxykinase (PEPCK) is the rate-limiting enzyme in gluconeogenesis and which catalyzes the glucose synthesis from metabolic precursors [39]. We found that *Pepck* mRNA expression levels significantly decreased in the 50 and 100 mg·kg^−1^ DHEA treatment groups. As PEPCK is one of the key enzymes of gluconeogenesis, we speculated that DHEA treatment inhibited the generation of glucose by reducing *Pepck* mRNA expression. Several studies have demonstrated that DHEA can promote the absorption of glucose in fibroblasts [40], adipocytes [41], muscle [42] and hepatocytes [43]. In some glucose-sensitive tissues, such as liver and muscle, the transfer of glucose depends on the transporter of GLUT-2 and GLUT-4 [44, 45]. The present study showed that *Glut2* mRNA expression levels in the 50 and 100 mg·kg^−1^ DHEA treatment groups, and *Glut4* mRNA expression level in the 50 mg·kg^−1^ DHEA treatment group significantly increased in the rats on a high-fat diet. This is consistent with a previous report by Sato *et al*., who found that DHEA activated GLUT-4 protein expression in skeletal muscles [42]. These results suggested that DHEA treatment could promote the absorption of serum glucose by increasing *Glut2* and *Glut4* mRNA expression levels.

Previous studies have shown that DHEA treatment enhanced the activities of the hexokinase and phosphofructokinase in the glycolytic pathway in the skeletal muscle cells of rats [42]. Thus, we postulate that DHEA might maintain the normal glucose levels by accelerating the glucose catabolism in rats on a high-fat diet. Our results showed that GK activities in the 50 and 100 mg·kg^−1^ DHEA treatment groups and PK activity in 100 mg·kg^−1^ DHEA treatment group were significantly enhanced in the liver of rats on a high-fat diet. The pyruvate dehydrogenase complex converts pyruvate into acetyl-CoA, which may then be used in the citric acid cycle for cellular respiration [46]. The reaction catalyzed by pyruvate dehydrogenase (E1) is considered to be the rate-limiting reaction in the pyruvate dehydrogenase complex [46, 47]. Our results also demonstrated that DHEA treatment significantly increased PDH (E1) activity in the liver of rats on a high-fat diet. SDH is a membrane-bound enzyme, which is the only enzyme that participates in both the citric acid cycle and the electron transport chain [48]. It has been shown that DHEA inhibits NAD-dependent mitochondrial respiration as well as Complex I in the mitochondrial respiratory chain [49], the consequences of which are ATP depletion, increased glucose uptake, and oxidization in the cells to compensate [50]. The present study demonstrated that DHEA-treatment (50 mg·kg^−1^) significantly increased SDH activity in the liver of rats on a high-fat diet. These results were similar to our previous study, which established that SDH activity increased in TM-3 cells after DHEA treatment [51]. In addition, MDH activity in the liver significantly increased in the 50 and 100 mg·kg^−1^ DHEA treatment groups. MDH is an enzyme that reversibly catalyzes the oxidation of malate to oxaloacetate; this reaction is a part of many metabolic pathways, including the citric acid cycle [52]. Taken together, the results indicate that DHEA may maintain normal glucose levels by enhancing some key enzyme activity to accelerate glucose catabolism in rats on a high-fat diet.

Hormones, especially insulin and glucagon, play important roles in controlling blood glucose levels in mammals. No changes were observed in glucagon content, whereas the 50 mg·kg^−1^ DHEA treatment significantly increased insulin content and insulin receptor (*Inrs*) mRNA expression levels in rats on a high-fat diet. Numerous studies have shown that DHEA treatment significantly increases serum insulin and leptin contents [23, 32]. Insulin is known to upregulate Akt phosphorylation *via* PI3K, which results in an increase in glucose uptake and utilization [24, 53]. Sato *et al*. reported that glucose metabolism-related signaling pathways and enzyme activity were enhanced after DHEA supplementation, and that they increased Akt and protein kinase C phosphorylation levels in skeletal muscles [42]. In addition, the 50 and 100 mg·kg^−1^ DHEA treatment significantly increased serum leptin content. Leptin and insulin play similar roles in the PI3K/Akt signaling pathway because the receptor substrate in both processes is insulin receptor substrate-2 (IRS-2) [54, 55]. When insulin binds to the insulin receptor (INSR), IRS-1 in muscle tissue and IRS-2 in liver tissue are activated, which subsequently activate the PI3K/Akt signaling pathway [56, 57]. Our findings indicated that the *Inrs*, *Irs2*, *Pi3k*, and *Akt* mRNA levels were significantly enhanced by DHEA administration in rats on a high-fat diet. These results indicated that DHEA treatment accelerate glucose catabolism may be associated with the activation of PI3K/Akt signaling pathway in rats fed high-fat diet. Akt promotes glucose transport and stimulates glycolysis through the activation of several glycolytic enzymes, including hexokinase and phosphofructokinase (PFK) [54, 58]. PFK-2 is the sole enzyme responsible for the production and degradation of fructose 2,6-bisphosphate, which allosterically activates PFK-1 more potently than its own product, fructose 1,6-bisphosphate [59]. In addition, PFK-2 may bind and directly activate GK to control the flux of fructose 6-phosphate substrate into fructose 1,6-bisphosphate [60, 61]. In this study, the 50 and 100 mg·kg^−1^ DHEA treatment significantly enhanced PFK-2 activity. As PFK-2 is one of the key enzymes of glycolysis, we speculated that treatment with DHEA activated the PI3K/Akt signaling pathway, which accelerated glucose catabolism by promoting PFK-2 activity.

**Fig 10:**
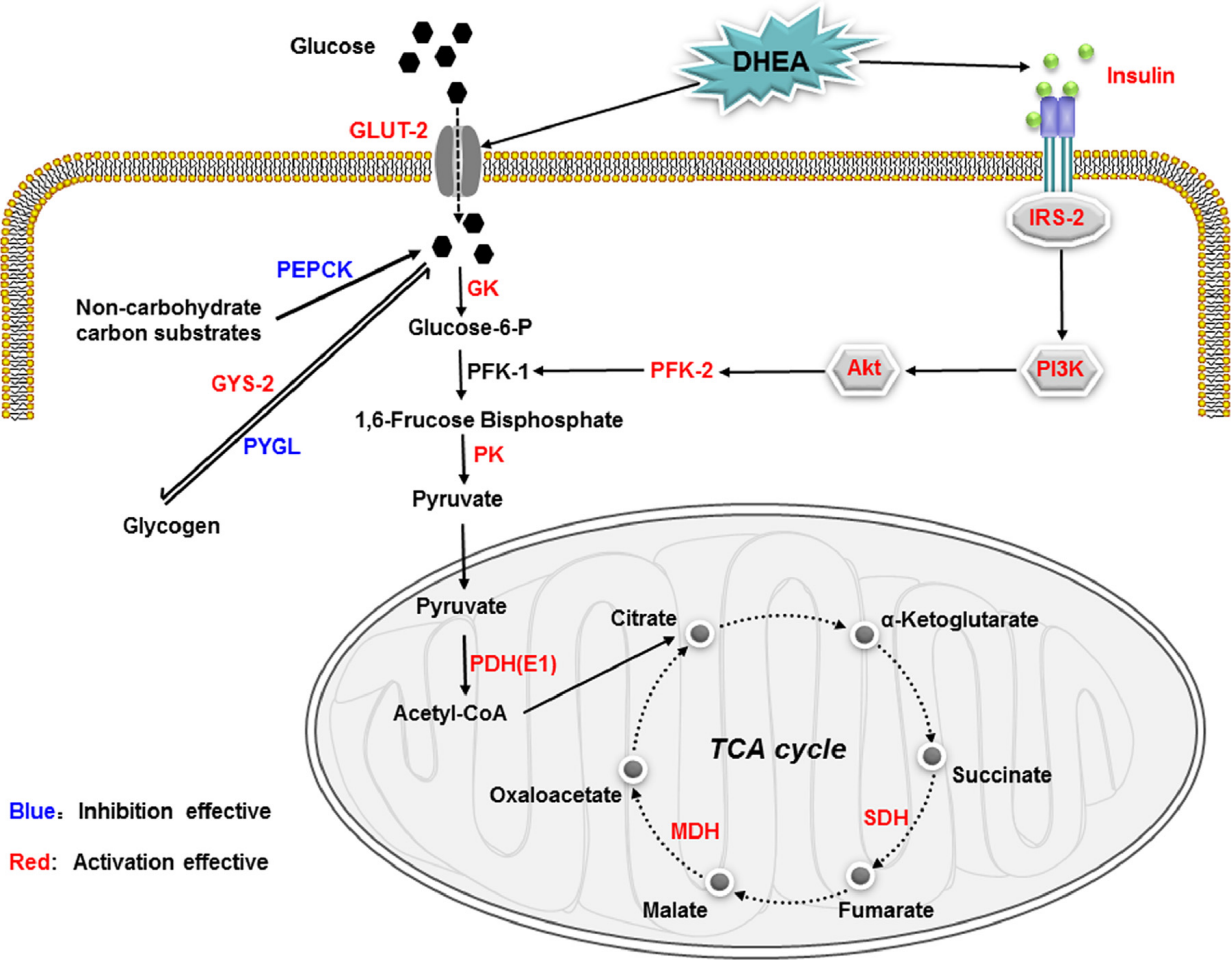
Schematic diagram of possible mechanisms of DHEA regulate glucose metabolism in rats fed a high-fat diet. Long-term DHEA administration could promote glycogen storage and accelerate glucose catabolism in rats on a high-fat diet. Notably, DHEA treatment accelerated glucose catabolism by promoting PFK-2 activity, and which may be associated with the activation of PI3K/Akt signaling pathway.

In conclusion (as shown in Fig 10), our findings demonstrated that DHEA treatment could promote glycogen storage and accelerate glucose catabolism in rats on a high-fat diet. Notably, DHEA treatment accelerated glucose catabolism by promoting PFK-2 activity in rats on a high-fat diet, which may be associated with the activation of PI3K/Akt signaling pathway. Certainly, further investigation should be focused on detecting the protein and phosphorylation levels, or blocking PI3K/Akt pathway to support this conclusion.

## Abbreviations

DHEA: dehydroepiandrosterone
BMI: body mass index
PDH: pyruvate dehydrogenase
*Pygl*: glycogen phosphorylase
PFK-2: phosphofructokinase-2
PK: pyruvate kinase
SDH: succinate dehydrogenase
MDH: malate dehydrogenase
GK: glucokinase
*Akt*: protein kinase B
*Glut2*: glucose transporter-2
*Glut4*: glucose transporter-4
*Irs1*: insulin receptor substrate-1
*Irs2*: insulin receptor substrate-2
*Pi3k*: phosphatidylinositol 3-kinase
*Gys2*: glycogen synthase-2
*Pepck*: phosphoenolpyruvate carboxykinase
*Inrs*: insulin receptor
NCG: normal diet control group
HCG: high-fat diet control group
HLG: high-fat diet with low dose group
HMG: high-fat diet with medium dose group
HHG: high-fat diet with high dose group

## References

[1] Kopelman PG. Obesity as a medical problem. Nature. 2000;404(6778):635–43. 10766250 10.1038/35007508

[2] Hernandez-Morante JJ, Cerezo D, Cruz RM, Larque E, Zamora S, Garaulet M. Dehydroepiandrosterone-sulfate modifies human fatty acid composition of different adipose tissue depots. Obes Surg. 2011;21(1):102–11. Epub 2010/01/23. 10.1007/s11695-009-0064-8 . 20094820

[3] Sturm R. Increases in morbid obesity in the USA: 2000–2005. Public Health. 2007;121(7):492–6. 10.1016/j.puhe.2007.01.006 . 17399752 PMC2864630

[4] Halimi S. Do not forget that type 2 diabetes does not only expose to cardiovascular complications. Diabetes & Metabolism. 2014;40(3):167–8. 24751987 10.1016/j.diabet.2014.03.005

[5] Kelly PJ, Clarke PM, Hayes AJ, Gerdtham UG, Cederholm J, Nilsson P, et al Predicting mortality in people with Type 2 diabetes mellitus after major complications: a study using Swedish National Diabetes Register data. Diabetic Medicine. 2014;31(8):954–62. 10.1111/dme.12468 . 24750341

[6] Hagiwara H, Kaizu K, Uriu K, Noguchi T, Takagi I, Qie YL, et al Expression of type-1 kidney plasminogen activator inhibitor in the of diabetic rat models. Thrombosis Research. 2003;111(4–5):301–9. 10.1016/j.thromres.2003.09.023 . 14693179

[7] Hariri N, Thibault L. High-fat diet-induced obesity in animal models. Nutrition Research Reviews. 2010;23(2):270–99. 10.1017/s0954422410000168 . 20977819

[8] Kim YJ, Kim K-Y, Kim MS, Lee JH, Lee KP, Park T. A mixture of the aqueous extract of Garcinia cambogia, soy peptide and L-carnitine reduces the accumulation of visceral fat mass in rats rendered obese by a high fat diet. Genes and Nutrition. 2008;2(4):353–8. 10.1007/s12263-007-0070-1 . 18850230 PMC2478482

[9] Roy S, Rink C, Khanna S, Phillips C, Bagchi D, Bagchi M, et al Body weight and abdominal fat gene expression profile in response to a novel hydroxycitric acid-based dietary supplement. Gene Expression. 2004;11(5–6):251–62. . 15200237 10.3727/000000003783992289 PMC5991152

[10] Adeneye AA, Adeyemi OO, Agbaje EO. Anti-obesity and antihyperlipidaemic effect of Hunteria umbellata seed extract in experimental hyperlipidaemia. Journal of Ethnopharmacology. 2010;130(2):307–14. 10.1016/j.jep.2010.05.009 . 20471465

[11] Karbowska J, Kochan Z. Fat-reducing effects of dehydroepiandrosterone involve upregulation of ATGL and HSL expression, and stimulation of lipolysis in adipose tissue. Steroids. 2012;77(13):1359–65. 10.1016/j.steroids.2012.08.002 . 22951290

[12] Liu GX, Han NN, Han J, Chen D, Kang J, Ma HT. Garcinia Cambogia extracts prevented fat accumulation via Adiponectin-AMPK signaling pathway in developing obesity rats. Food Science and Technology Research. 2015;21(6):835–45.

[13] Suge R, Shimazu T, Hasegawa H, Inoue I, Hayashibe H, Nagasaka H, et al Cerebral antioxidant enzyme increase associated with learning deficit in type 2 diabetes rats. Brain Research. 2012;1481:97–106. 10.1016/j.brainres.2012.08.056 . 22981416

[14] Monette MCE, Baird A, Jackson DL. A Meta-Analysis of Cognitive Functioning in Nondemented Adults with Type 2 Diabetes Mellitus. Canadian Journal of Diabetes. 2014;38(6):401–8. 10.1016/j.jcjd.2014.01.014 . 24933107

[15] Naredi N, Sandeep K, Jamwal VDS, Nagraj N, Rai S. Dehydroepiandrosterone: A panacea for the ageing ovary? Medical journal, Armed Forces India. 2015;71(3):274–7. 10.1016/j.mjafi.2014.12.022 . PMC4534532 26288496

[16] Goel RM, Cappola AR. Dehydroepiandrosterone sulfate and postmenopausal women. Current Opinion in Endocrinology Diabetes and Obesity. 2011;18(3):171–6. 10.1097/MED.0b013e3283461818 . 21478748

[17] Bacsi K, Kosa J, Lazary A, Horvath H, Balla B, Lakatos P, et al Significance of dehydroepiandrosterone and dehydroepiandrosterone sulfate in different diseases. Orvosi hetilap. 2007;148(14):651–7. 10.1556/oh.2007.27903 . 17403638

[18] Jose Hernandez-Morante J, Cerezo D, Maria Cruz R, Larque E, Zamora S, Garaulet M. Dehydroepiandrosterone-Sulfate Modifies Human Fatty Acid Composition of Different Adipose Tissue Depots. Obesity Surgery. 2011;21(1):102–11. 10.1007/s11695-009-0064-8 . 20094820

[19] Legrain S, Girard L. Pharmacology and therapeutic effects of dehydroepiandrosterone in older subjects. Drugs & Aging. 2003;20(13):949–67. 10.2165/00002512-200320130-00001 . 14561100

[20] Karbowska J, Kochan Z. Effects of DHEA on metabolic and endocrine functions of adipose tissue. Hormone molecular biology and clinical investigation. 2013;14(2):65–74. 10.1515/hmbci-2013-0009 . 25436721

[21] Tang X, Ma H, Shen Z, Zou S, Xu X, Lin C. Dehydroepiandrosterone activates cyclic adenosine 3',5'-monophosphate/protein kinase A signalling and suppresses sterol regulatory element-binding protein-1 expression in cultured primary chicken hepatocytes. British Journal of Nutrition. 2009;102(5):680–6. 10.1017/S0007114509289021 19267949

[22] Tang X, Ma H, Zou S, Chen W. Effects of dehydroepiandrosterone (DHEA) on hepatic lipid metabolism parameters and lipogenic gene mRNA expression in broiler chickens. Lipids. 2007;42(11):1025–33. 10.1007/s11745-007-3104-y . 17704960

[23] Zhou Y, Kang J, Chen D, Han N, Ma H. Ample Evidence: Dehydroepiandrosterone (DHEA) Conversion into Activated Steroid Hormones Occurs in Adrenal and Ovary in Female Rat. Plos One. 2015;10(5). 10.1371/journal.pone.0124511 PMC4427309 25962158

[24] Song L, Tang X, Kong Y, Ma H, Zou S. The expression of serum steroid sex hormones and steroidogenic enzymes following intraperitoneal administration of dehydroepiandrosterone (DHEA) in male rats. Steroids. 2010;75(3):213–8. 10.1016/j.steroids.2009.11.007 19961867

[25] Cleary MP. THE ANTIOBESITY EFFECT OF DEHYDROEPIANDROSTERONE IN RATS. Proceedings of the Society for Experimental Biology and Medicine. 1991;196(1):8–16. . 1824576 10.3181/00379727-196-43158b

[26] Gansler TS, Muller S, Cleary MP. CHRONIC ADMINISTRATION OF DEHYDROEPIANDROSTERONE REDUCES PANCREATIC BETA-CELL HYPERPLASIA AND HYPERINSULINEMIA IN GENETICALLY-OBESE ZUCKER RATS. Proceedings of the Society for Experimental Biology and Medicine. 1985;180(1):155–62. . 3162168 10.3181/00379727-180-42158

[27] Chen J, Tang X, Zhang Y, Ma H, Zou S. Effects of maternal treatment of dehydroepiandrosterone (DHEA) on serum lipid profile and hepatic lipid metabolism-related gene expression in embryonic chickens. Comparative Biochemistry and Physiology B-Biochemistry & Molecular Biology. 2010;155(4):380–6. 10.1016/j.cbpb.2009.12.005 . 20060923

[28] Zhang Y, Chen J, Ma H, Zou S. Effects of Maternal Dietary Treatment with Dehydroepiandrosterone on Lipid Metabolism Parameters in Offspring Broilers. Journal of Animal and Veterinary Advances. 2012;11(23):4332–9. .

[29] Cleary MP, Zisk JF. ANTIOBESITY EFFECT OF 2 DIFFERENT LEVELS OF DEHYDROEPIANDROSTERONE IN LEAN AND OBESE MIDDLE-AGED FEMALE ZUCKER RATS. International Journal of Obesity. 1986;10(3):193–204. . 2944850

[30] Mauriege P, Martel C, Langin D, Lacaille M, Despres JP, Belanger A, et al Chronic effects of dehydroepiandrosterone on rat adipose tissue metabolism. Metabolism-Clinical and Experimental. 2003;52(3):264–72. 10.1053/meta.2003.50043 . 12647261

[31] Karbowska J, Kochan Z. Effect of DHEA on endocrine functions of adipose tissue, the involvement of PPAR gamma. Biochemical Pharmacology. 2005;70(2):249–57. 10.1016/j.bcp.2005.04.022 . 15904896

[32] Sato K, Iemitsu M, Aizawa K, Mesaki N, Ajisaka R, Fujita S. DHEA administration and exercise training improves insulin resistance in obese rats. Nutrition & Metabolism. 2012;9 10.1186/1743-7075-9-47 . PMC3433349 22647230

[33] Villareal DT, Holloszy JO. Effect of DHEA on abdominal fat and insulin action in elderly women and men—A randomized controlled trial. Jama-Journal of the American Medical Association. 2004;292(18):2243–8. 10.1001/jama.292.18.2243 . 15536111

[34] De Pergola G. The adipose tissue metabolism: role of testosterone and dehydroepiandrosterone. International Journal of Obesity. 2000;24:S59–S63. . 10997611 10.1038/sj.ijo.0801280

[35] Mayer D, Reuter S, Hoffmann H, Bocker T, Bannasch P. Dehydroepiandrosterone reduces expression of glycolytic and gluconeogenic enzymes in the liver of male and female rats. International Journal of Oncology. 1996;8(6):1069–78. . 21544466 10.3892/ijo.8.6.1069

[36] Agius L. Role of glycogen phosphorylase in liver glycogen metabolism. Molecular aspects of medicine. 2015;46:34–45. 10.1016/j.mam.2015.09.002 . 26519772

[37] Farkas I, Hardy TA, Goebl MG, Roach PJ. 2 GLYCOGEN-SYNTHASE ISOFORMS IN SACCHAROMYCES-CEREVISIAE ARE CODED BY DISTINCT GENES THAT ARE DIFFERENTIALLY CONTROLLED. Journal of Biological Chemistry. 1991;266(24):15602–7. . 1908457

[38] Nelson DL, Lehninger AL, Cox MM. Lehninger principles of biochemistry. Worth Publishers. 2013;32(10):947–8.

[39] Chakravarty K, Cassuto H, Reshef L, Hanson RW. Factors That Control the Tissue-Specific Transcription of the Gene for Phosphoenolpyruvate Carboxykinase-C. Critical Reviews in Biochemistry & Molecular Biology. 2005;40(3):129–54(26). 15917397 10.1080/10409230590935479

[40] Nakashima N, Haji M, Sakai Y, Ono Y, Umeda F, Nawata H. Effect of dehydroepiandrosterone on glucose uptake in cultured human fibroblasts. Metabolism Clinical & Experimental. 1995;44(4):543–8. 7723680 10.1016/0026-0495(95)90065-9

[41] Sebastio P, Annalisa N, Luigi L, Gaetana B, Carmela M, Angelo C, et al Dehydroepiandrosterone stimulates glucose uptake in human and murine adipocytes by inducing GLUT1 and GLUT4 translocation to the plasma membrane. Diabetes. 2004;53(1):41–52. 14693696 10.2337/diabetes.53.1.41

[42] Sato K, Iemitsu M, Aizawa K, Ajisaka R. Testosterone and DHEA activate the glucose metabolism-related signaling pathway in skeletal muscle. Ajp Endocrinology & Metabolism. 2008;294(5). 10.1152/ajpendo.00678.2007 18349113

[43] Yamashita R, Saito T, Satoh S, Aoki K, Kaburagi Y, Sekihara H. Effects of dehydroepiandrosterone on gluconeogenic enzymes and glucose uptake in human hepatoma cell line, HepG2. Endocrine Journal. 2005;52(6):727–33. 16410665 10.1507/endocrj.52.727

[44] Marie S, Diazguerra MJ, Miquerol L, Kahn A, Iynedjian PB. THE PYRUVATE-KINASE GENE AS A MODEL FOR STUDIES OF GLUCOSE-DEPENDENT REGULATION OF GENE-EXPRESSION IN THE ENDOCRINE PANCREATIC BETA-CELL TYPE. Journal of Biological Chemistry. 1993;268(32):23881–90. . 8226928

[45] Rencurel F, Waeber G, Antoine B, Rocchiccioli F, Maulard P, Girard J, et al Requirement of glucose metabolism for regulation of glucose transporter type 2 (GLUTP) gene expression in liver. Biochemical Journal. 1996;314:903–9. . 8615787 10.1042/bj3140903 PMC1217142

[46] Ciszak EM, Korotchkina LG, Dominiak PM, Sukhdeep S, Patel MS. Structural basis for flip-flop action of thiamin pyrophosphate-dependent enzymes revealed by human pyruvate dehydrogenase. Journal of Biological Chemistry. 2003;278(23):21240–6. 12651851 10.1074/jbc.M300339200

[47] Jaimes R, Kuzmiak-Glancy S, Brooks DM, Swift LM, Posnack NG, Kay MW. Functional response of the isolated, perfused normoxic heart to pyruvate dehydrogenase activation by dichloroacetate and pyruvate. Pflügers Archiv—European Journal of Physiology. 2015:1–12. 10.1007/s00424-015-1717-1 PMC4701640 26142699

[48] Rutter J, Winge DR, Schiffman JD. Succinate dehydrogenase—Assembly, regulation and role in human disease. Mitochondrion. 2010;10(4):393–401. 10.1016/j.mito.2010.03.001 20226277 PMC2874626

[49] Safiulina D, Peet N, Seppet E, Zharkovsky A, Kaasik A. Dehydroepiandrosterone inhibits complex I of the mitochondrial respiratory chain and is neurotoxic in vitro and in vivo at high concentrations. Toxicological Sciences An Official Journal of the Society of Toxicology. 2006;93(2):348–56. 16849397 10.1093/toxsci/kfl064

[50] Yang NC, Jeng KC, Ho WM, Hu ML. ATP depletion is an important factor in DHEA-induced growth inhibition and apoptosis in BV-2 cells. Life Sciences. 2002;70(17):1979–88. 12148690 10.1016/s0024-3205(01)01542-9

[51] Shen X, Lin L, Yin F, Ma H, Zou S. Effect of dehydroepiandrosterone on cell growth and mitochondrial function in TM-3 cells. General & Comparative Endocrinology. 2012;177(1):177–86. 22465782 10.1016/j.ygcen.2012.03.007

[52] Minárik P, Tomásková N, Kollárová M, Antalík M. Malate dehydrogenases—structure and function. General Physiology & Biophysics. 2002;21(3):257–65. 12537350

[53] Jessen N, Pold R, Buhl ES, Jensen LS, Schmitz O, Lund S. Effects of AICAR and exercise on insulin-stimulated glucose uptake, signaling, and GLUT-4 content in rat muscles. J Appl Physiol 94:1373–1379. Journal of Applied Physiology. 2003;94(4):1373–9. 12496137 10.1152/japplphysiol.00250.2002

[54] Gui S, Gang Y, Lu W, Zhou L, Ying X, Yu Y, et al Wnt3a regulates proliferation, apoptosis and function of pancreatic NIT-1 beta cells via activation of IRS2/PI3K signaling. Journal of Cellular Biochemistry. 2013;114(7):1488–97. 10.1002/jcb.24490 23296977

[55] Kim Y, Uotani S, Dd, Flier J, Kahn B. In vivo administration of leptin activates signal transduction directly in insulin-sensitive tissues: overlapping but distinct pathways from insulin. Endocrinology. 2000;141(7):2328–39. 10875232 10.1210/endo.141.7.7536

[56] Kubota N, Kubota T, Itoh S, Kumagai H, Kozono H, Takamoto I, et al Dynamic Functional Relay between Insulin Receptor Substrate 1 and 2 in Hepatic Insulin Signaling during Fasting and Feeding. Cell Metabolism. 2008;8(1):49–64. 10.1016/j.cmet.2008.05.007 18590692

[57] Sara FJ, Christine D, Laurent M, Carey AL, Assam EO, Kingwell BA, et al Phosphoinositide 3-kinase as a novel functional target for the regulation of the insulin signaling pathway by SIRT1. Molecular & Cellular Endocrinology. 2011;335(2):166–76. 21241768 10.1016/j.mce.2011.01.008

[58] Lunt SY, Heiden MGV. Aerobic glycolysis: meeting the metabolic requirements of cell proliferation. Annual Review of Cell & Developmental Biology. 2011;27(27):441–64. 10.1146/annurev-cellbio-092910-154237 21985671

[59] Burch PT, Berner DK, Najafi H,., M D M, Matschinsky FM. Regulatory role of fructose-2,6-bisphosphate in pancreatic islet glucose metabolism remains unsettled. Diabetes. 1985;34(10):1014–8. 3899804 10.2337/diab.34.10.1014

[60] Merrins MJ, Dyke AR, Van, Mapp AK, Rizzo MA, Satin LS. Direct Measurements of Oscillatory Glycolysis in Pancreatic Islet β-Cells Using Novel Fluorescence Resonance Energy Transfer (FRET) Biosensors for Pyruvate Kinase M2 Activity Journal of Biological Chemistry. 2013;288(46):33312–22. 10.1074/jbc.M113.508127 24100037 PMC3829177

[61] Merrins MJ, Bertram R, Sherman A, Satin LS. Phosphofructo-2-kinase/Fructose-2,6-bisphosphatase Modulates Oscillations of Pancreatic Islet Metabolism. Plos One. 2012;7(4):: e34036 10.1371/journal.pone.0034036 22532827 PMC3332096

